# Public involvement and engagement in big data research: A scoping review

**DOI:** 10.23889/ijpds.v8i2.2247

**Published:** 2023-09-14

**Authors:** Piotr Teodorowski, Elisa Jones, Saiqa Ahmed, Naheed Tahir, Lucy Frith

**Affiliations:** 1 University of Liverpool, Liverpool, United Kingdom; 2 NIHR ARC NWC, Liverpool, United Kingdom; 3 University of Manchester, Manchester, United Kingdom

## Objectives

Public involvement and engagement have been suggested as a way to establish public support for big data research, yet there has been no review exploring how these activities could facilitate this. Therefore, this scoping review aimed to explore public involvement and engagement in big data research.

## Method

Following Arksey and O’Malley’s methodology, we systematically searched the following databases: CINAHL, Health Research Premium Collection, PubMed, Scopus and Web of Science for papers published between 2010-2021. Additional manual searches took place. These included the first 100 hits in Google search, journals (BMC Research Involvement and Engagement, International Journal of Population Data Science and Health Expectations) and grey literature (Patient Outcome Research Institute database, first 100 hits were screened). We extracted data using a standardised form. We then organised it in a descriptive and narrative way. A system logic model was developed to understand the complexity of this topic.

## Results

Fifty-three papers were identified as eligible for inclusion in our review. The findings indicate that public involvement and engagement have the potential to improve public trust and accountability for data resharing for research. However, there is limited literature actually evaluating these activities. The findings suggest that the public can be meaningfully involved and engaged in big data research, both in terms of individual research projects and data governance, but there is no one standardised approach to do it. Therefore, we developed an initial system logic model to map relevant aspects of the involvement and engagement activities. These include which communities to reach, the context (e.g. ethical, legal aspects or public views), the design and delivery of activities, and outcomes.

## Conclusion

Despite the growing literature on public involvement and engagement in big data research, more research is needed as there are few primary empirical studies exploring involvement and engagement. We suggest using the system logic model we developed when reflecting on issues that might be relevant in organising these activities.

